# Probiotic *Escherichia coli* Ameliorates Antibiotic-Associated Anxiety Responses in Mice

**DOI:** 10.3390/nu13030811

**Published:** 2021-03-01

**Authors:** Kiwoong Park, Suhyeon Park, Arulkumar Nagappan, Navin Ray, Juil Kim, Sik Yoon, Yuseok Moon

**Affiliations:** 1 Laboratory of Mucosal Exposome and Biomodulation, Department of Integrative Biomedical Sciences, Biomedical Research Institute, Pusan National University, Yangsan 50612, Korea; keroro56@daum.net (K.P.); cutedina16@naver.com (S.P.); arulbiotechtnau@gmail.com (A.N.); navin.ray@gmail.com (N.R.); 1022myths@hanmail.net (J.K.); 2 Department of Medicine, Pusan National University, Yangsan 50612, Korea; 3 Department of Anatomy, College of Medicine, Pusan National University, Yangsan 50612, Korea; sikyoon@pusan.ac.kr

**Keywords:** antibiotics, streptomycin, probiotics, anxiety

## Abstract

Despite the beneficial actions of antibiotics against bacterial infections, the use of antibiotics is a crucial etiological factor influencing microbial dysbiosis-associated adverse outcomes in human health. Based on the assumption that gut microbial dysbiosis can provoke behavioral or psychological disorders, the present study evaluated anxiety-linked behavioral changes in a mouse model of streptomycin-induced dysbiosis. Measuring anxiety-like behavior using the light–dark box and elevated plus maze tests indicated that streptomycin treatment caused acute anxiety in mice. As an intervention for dysbiosis-associated distress, the probiotic strain *Escherichia coli* Nissle 1917 (EcN) was evaluated for its effects on streptomycin-induced behavioral changes in mice. EcN supplementation persistently ameliorated anxiety responses in mice with streptomycin-induced dysbiosis. As an outcome of anxiety, body weight changes were marginally affected by antibiotic treatment. However, mice supplemented with EcN displayed acute retardation of body weight gain, since EcN is known to reduce food intake and increase energy expenditure. Taken together, EcN treatment prominently counteracted streptomycin-induced anxiety in mice, with the metabolically beneficial retardation of body weight gain. The present model simulates psychological disorders in antibiotic users. As a promising intervention, EcN treatment can facilitate psychological relief under conditions of dysbiotic stress by blocking the pathologic gut–brain circuit.

## 1. Introduction

Aminoglycoside antibiotics, including streptomycin, are natural or semisynthetic antibiotics derived from actinomycetes. With regard to the rise of multidrug-resistant bacteria, aminoglycoside antibiotics are used in optimized dosing regimens as broad-spectrum rapid bactericidal agents [1]. Despite its beneficial action against infection, streptomycin poses potential risks, such as kidney and ear toxicity; the latter may lead to transient, permanent, or congenital deafness in maternally exposed children. Moreover, other side effects include vertigo, vomiting, numbness of the face, fever, and rash from persistent use [2]. Streptomycin is highly effective against gram-negative aerobes and some anaerobic bacilli, whereas it generally lacks bactericidal activity against gram-positive and anaerobic gram-negative bacteria [1]. Owing to this antibacterial action, it can cause an imbalance of the bacterial community in the gut, which is called dysbiosis [3,4]; this may be associated with subsequent disease outcomes or complications.

The gut microbiota (GM) plays a key role in modulating the development and functioning of various physiological processes, including homeostasis maintenance [5]. *Escherichia coli* strain Nissle 1917 (O6:K5:H1, EcN), a non-pathogenic fecal bacterium, exerts probiotic effects in humans and animals [6,7,8]. This strain was originally isolated from the gut of a German soldier who, unlike his comrades, displayed resistance to endemic shigellosis during World War I [9]. EcN does not produce any virulence factors, carry any genes for pathogenicity traits, or form enterotoxins, cytotoxins, or hemolysins [10,11]. Moreover, EcN treatment can counteract infectious diseases, such as mucosal distress from *Salmonella* or enteropathogenic *E. coli*, via the synthesis of barrier-linked molecules, including defensins and zonula occludens protein-1 [12,13]. Therefore, EcN has been increasingly implicated as a potential agent that could be used for the intervention of dysbiosis during inflammation and tumorigenesis in the mucosal niche [14]. EcN showed better inhibition of the growth of *E. coli* than any other commensal *E. coli* strains in the intestines of streptomycin-treated mice [15,16,17]. Limited clinical investigations using EcN have demonstrated that probiotic-based therapeutic applications can be efficacious in patients with chronic ulcerative colitis [18,19,20] and irritable bowel syndrome [21]. EcN is relatively safe for therapeutic applications because it does not cause colitis, even in gnotobiotic animals that are mono-inoculated with the strain [14,22].

Dysbiosis is closely associated with multiple chronic diseases, including obesity, asthma, and several chronic inflammatory diseases [5,23]. Recent reports have suggested that the GM communicates with the neuronal system via the gut–brain axis, which involves neural pathways functioning through the vagus nerve, and the endocrine and immune system pathways [24,25,26,27,28]. Detrimental alterations in the GM community have crucial impacts on neurogenerative processes and have been associated with neuropsychiatric disorders, such as autism, schizophrenia, and anxiety and depressive disorders [29,30]. The use of antibiotics is the primary etiological factor that alters GM diversity and may thus, cause adverse health effects. A population-based study has reported that specific antibiotic exposure is significantly correlated with a higher risk for depression and anxiety [31]. In particular, prenatal or early life antibiotic exposure is associated with an increased risk for psychiatric disorders [32], which is consistent in the experimental animal model of microbiota depletion by chronic postweaning exposure to antibiotics [32]. At the molecular levels, human population-based assessment of gene-environment interaction indicated the positive associations of early-life long-term exposure to antibiotics with the expression of genes involved in anxiety and depression [33]. Moreover, antibiotic-induced behavioral alterations have been extensively evaluated in association with neuroendocrine regulation in diverse experimental models [34,35,36]. In contrast, a meta-analysis and experimental animal-based assessments demonstrated that supplementation with probiotics counteracts dysbiosis and attenuates levels of depression or anxiety variables [37,38,39]. Hence, we hypothesized that antibiotic-induced dysbiosis can provoke behavioral or psychological disorders. Experimental mice were exposed to streptomycin to disrupt the gut microbial community, and we evaluated the effects of EcN as a potential intervention against antibiotic-induced distress.

## 2. Materials and Methods

### 2.1. Animal Experiments

**Figure 1:**
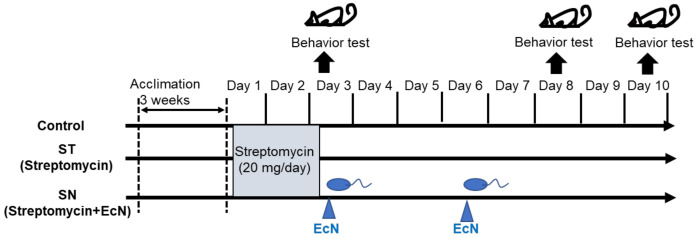
Schematic diagram of the experimental study design. Male C57BL/6 mice were divided into three groups (*n* = 7 per group): control, ST (Streptomycin-treated group), and SN (Streptomycin and EcN-treated group). On day 1, streptomycin (20 mg/day) was administered to the mice from both ST and SN groups. Only the mice in the SN group were administered EcN (1 × CFU) via oral gavage on days 3 and 6. Behavior tests were conducted on days 3, 8, and 10. Day 3 behavior tests were performed before EcN treatment. Body weight was also measured on days 3 and 10 (EcN: *Escherichia coli* strain Nissle 1917; CFU: colony-forming units).

In total, six-week-old C57BL/6 mice (weighing 16–18 g on average) were purchased from Jackson Laboratories (Bar Harbor, ME, USA). The mice were sufficiently acclimated for 21 days before the experiments and maintained at 25 °C in 45–55% relative humidity under 12 h light–dark cycles. Male C57BL/6 mice were divided into three groups (*n* = 7 per group): control, ST (Streptomycin-treated group), and SN (Streptomycin and EcN-treated group). They were housed two or three per cage and provided with sufficient food and water in environmentally protected cages comprising a transparent polypropylene body and a stainless-steel wire top cover. The design and procedure of the animal experiments are presented as a schematic diagram in Figure 1. For antibiotic treatment, the mice were administered 2 g/L streptomycin (Sigma–Aldrich, St. Louis, MO, USA) in drinking water for 48 h, which corresponds to 20 mg per mouse per day. For probiotic treatment, EcN was kindly provided by Ardeypharm GmbH (Herdecke, Germany). The strain was routinely grown at 37 °C in Luria–Bertani medium to an optical density at 600 nm (OD_600_) of approximately 1, washed by centrifugation, and resuspended in serum-free Roswell Park Memorial Institute medium. For probiotic treatment, the mice were intragastrically gavaged twice with 0.2 mL of a suspension containing 10^9^ colony-forming units (cfu)/mL of EcN at 72 h intervals after antibiotic treatment. Based on the pharmacological dose in 35–84 day human trials (0.5–1 × 10^11^ cfu/day) [40,41], total extrapolated loads in mice can be about 1–3 × 10^10^ cfu with consideration of body weight and frequency. For safety in acute exposure, the actual treatment dose was reduced to 2 × 10^9^ cfu which corresponds to one-tenth of the total extrapolated levels. Mice were twice exposed via gavage with 10^9^ cfu/0.2 mL [14].

### 2.2. Behavior Tests for Anxiety

Behavior tests were conducted on days 3 (Before EcN treatment), 8, and 10. The light–dark box (LDB) and elevated plus maze (EPM) tests were conducted to test for anxiety in mice [42,43]. The LDB test was performed in a light–dark box apparatus with dimensions of 50 × 25 × 25 cm, which consisted of two chambers made of black and white Styrofoam boards that were linked by an opening (7.5 × 7.5 cm) located at the floor level in the center of the dividing wall. The test was performed on each mouse for 3 min. Each mouse was placed in the dark chamber and allowed to freely explore the apparatus. Time spent in the dark and light compartments was measured and used as indices of anxiety. Longer time durations spent in the light chamber translated to lower anxiety levels for each mouse. The EPM test was performed in a plus-maze apparatus, which consisted of two open (22 × 7 cm) and two enclosed arms (22 × 7 cm) with 22 cm high walls extending from a central platform (7 × 7 cm) on a 50 cm tall support column. The arms, walls, and arm supporting columns were made of Styrofoam boards; the main column supporting the central platform was made of hardboard papers. The test was performed on each mouse for 3 min. Each mouse was first placed on the central platform and allowed to explore the four arms in each direction. Time spent in the open arms was measured (time at the central platform was selectively counted as the time spent in the open arms; the time for which the mouse was touching, or its head was exploring, the open arms, was counted even though the lower half of the mouse’s body was at the central platform). Longer durations of time spent on the open arms translated to lower anxiety levels for each mouse. The body weight of mice was measured with the electric weighing balance on day 3 and 10. The values of the body weight were converted into percentiles by comparing with the average body weight of mice in the day 3 control group.

### 2.3. Statistical Analysis

All statistical analyses were performed using GraphPad Prism 5.04 software (GraphPad Software, San Diego, CA, USA). Data analysis was performed using a one-way analysis of variance, and the Tukey–Kramer post-hoc test was used to determine differences between treatment groups. All data presented in this study are expressed as the means ± standard deviations. Differences with *p* values < 0.05 were considered to be statistically significant.

## 3. Results

### 3.1. Streptomycin Treatment Causes Acute Anxiety in Mice

**Figure 2:**
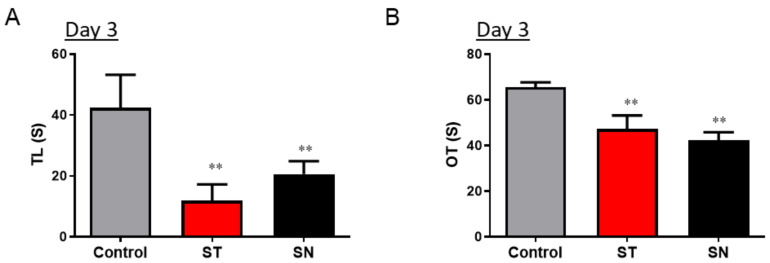
Treatment with streptomycin causes anxiety in C57BL/6 mice. Streptomycin (20 mg/day) was administered to mice from both the experimental groups on day 1. The LDB (**A**) and EPM (**B**) tests were conducted on day 3 after streptomycin treatment to study anxiety-like behaviors, ** *p* < 0.01 for control vs. ST and SN mice. (LDB: light-dark box; EPM: elevated plus maze; TL: time spent in light chamber (in seconds); OT: time spent in open arms (in seconds); ST: streptomycin alone-treated group; SN: streptomycin- and EcN-treated group).

Dysbiosis and inflammation in the gut have been associated with several mental illnesses, such as anxiety and depression [44]. In the present study, streptomycin was administered to C57BL/6 mice, and their behavior was evaluated to determine whether antibiotic-induced dysbiosis can affect anxiety responses and psychological disorders. Anxiety-like behavior was assessed in the mouse model of streptomycin-induced dysbiosis before probiotic treatment (Figure 1). The LDB test demonstrated that exposure to streptomycin significantly decreased the duration of staying in the light chamber in the ST (*p* = 0.001) and SN groups (*p* = 0.009) (Figure 2A) compared to that in the control group. The EPM test also revealed that streptomycin exposure significantly decreased the time spent in the open arms in the ST (*p* = 0.0004) and SN groups (*p* = 0.0006) (Figure 2B). These results clearly indicate that streptomycin treatment causes acute anxiety in C57BL/6 mice, which was then further assessed by EcN treatment.

### 3.2. EcN Supplementation Alleviates Streptomycin-Induced Anxiety in Mice

**Figure 3:**
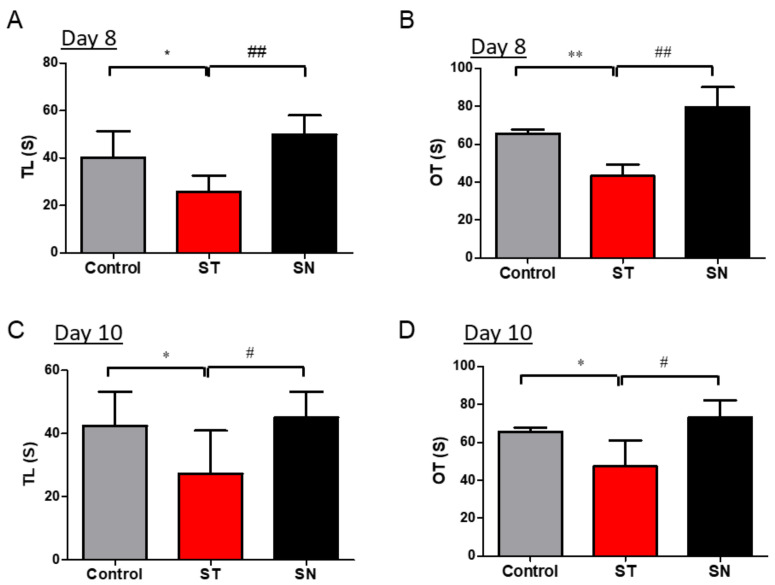
Treatment with EcN alleviates streptomycin-induced anxiety in C57BL/6 mice. Streptomycin (20 mg/day) was administered to mice from both ST and SN groups on day 1, and only the mice from SN were treated with EcN (1 × CFU) via oral gavage on days 3 and 6. The LDB (**A**,**C**) and EPM (**B**,**D**) tests were conducted on day 8 and day 10 to study anxiety-like behaviors. Data are expressed as the means ± SDs (*n* = 7). * *p* < 0.05 and ** *p* < 0.01 for control vs. ST mice; ^#^ *p* < 0.05 and ^# #^ *p* < 0.01 for ST vs. SN mice. (EcN: *Escherichia coli* strain Nissle 1917; CFU: colony-forming units; LDB: light-dark box; EPM: elevated plus maze; TL: time spent in light chamber (in seconds); OT: time spent in open arms (in seconds); ST: streptomycin alone-treated group; SN: streptomycin- and EcN-treated group; SD: standard deviation)

Based on the assumption that probiotic treatment can counteract dysbiosis-associated disorders such as anxiety, EcN supplementation was evaluated for its effects on streptomycin-induced behavioral changes in mice. The mice were subjected to the dual administration of EcN, which approximately corresponds to the level efficacious in humans, as recommended by the pharmaceutical manufacturer [40,41]. Anxiety-like behaviors were assessed using the LDB and EPM tests at 48 h (day 8) after the second oral gavage of EcN. Mice treated with EcN (SN group) displayed significant recovery from streptomycin-induced anxiety (*p* = 0.000014) compared to those from the ST group, which were administered streptomycin in the absence of the probiotic bacteria (Figure 3A,B). Mice in the ST group tended to spend less time in the light chamber and open arms in the LDB (Figure 3A) and EPM (Figure 3B) tests, respectively. In contrast, the mice from the SN group stayed in the light chamber and open arms for longer time durations than those from the ST group and even those from the control group.

Behavior tests were performed again at 96 h (day 10) after the second EcN treatment to observe whether there were any changes in, or the attenuation of, the effects of EcN or streptomycin treatment over time (Figure 3C,D). The results of both the LDB and EPM tests on day 10 were quite similar to those obtained on day 8 (Figure 3A,B). Streptomycin-induced anxiety was persistently observed in the ST group on day 10 (Figure 3C,D). Moreover, EcN-induced attenuation of anxiety was still observed in the mice from the SN group, although there was a reduction in probiotic-induced anti-anxiety effects over time. Taken together, EcN supplementation persistently ameliorated the anxiety responses in mice with streptomycin-induced dysbiosis.

**Figure 4:**
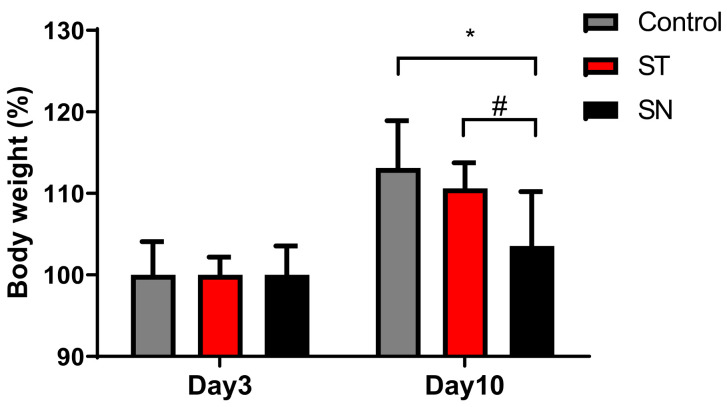
Treatment with EcN prevents body weight gain in C57BL/6 mice. Streptomycin (20 mg/day) was administered to mice in both ST and ST groups on day 1, and only the mice from SN were treated with EcN (1 × CFU) via oral gavage on days 3 and 6. The body weight of each mouse was measured on days 3 and 10. Data are expressed as the means ± SDs (*n* = 7). * *p* < 0.05 vs. control mice and ^#^ *p* < 0.05 vs. ST mice. (EcN: *Escherichia coli* strain Nissle 1917; CFU: colony-forming units; ST: streptomycin alone-treated group; SN: streptomycin- and EcN-treated group; SD: standard deviation)

As an outcome of anxiety, body weight changes were assessed, as there are known associations between anxiety and body weight changes [45,46,47]. The average body weight of the mice from the control, ST, and SN groups were converted into percentiles to compare the progression of body weight gain following antibiotic and probiotic treatment (Figure 4). After 10 days, mice in the ST group with elevated streptomycin-induced anxiety did not display significant body weight changes. However, the mice from the SN group showed a significantly reduced gain in body weight compared to those from the control (*p* = 0.0243) and ST groups (*p* = 0.0407). Thus, EcN supplementation retarded body weight gain in C57BL/6 mice with streptomycin-induced anxiety.

## 4. Discussion

The present study aimed to investigate the psychological influence of GM on the central nervous system after the streptomycin treatment of mice. Antibiotics have been used to treat or prevent bacterial infections, but sometimes they cause severe life-threatening side effects that increase the economic burden and mortality of the global population [48,49]. The main problem with antibiotics is that they do not fight against specific harmful bacteria, but instead, kill a variety of other beneficial microorganisms in the gut. In the present study, streptomycin-induced disturbances in the GM mediated psychological shifts in mice, which was counteracted by EcN, which acted as a potent intervention against dysbiosis-linked distress. Dysbiosis or imbalance in the gut microbial community has been associated with several mental illnesses, such as anxiety and depression [44]. Many clinical studies have reported that antibiotics can aggravate the imbalance of the microbial community in the intestines [50,51,52,53]. Moreover, the frequent use of antibiotics may also increase the risks of psychological disorders in the human population [29,30,31]. In the present study, streptomycin appeared to cause severe anxiety-linked responses, as assessed using both the LDB and EPM tests (Figure 2); these responses persisted for one week after streptomycin treatment in mice. Similar to our results, specific antibiotic exposure has been reported to be associated with a higher risk for depression and anxiety, but not psychosis [31]. Since the effect of antibiotics on psychological status is not just momentary, extended observation of animals subjected to acute antibiotic exposure or the analysis of the effects of repetitive antibiotic treatment are warranted to address the comprehensive impacts of streptomycin on anxiety.

The microbiota and their corresponding metabolites communicate with the host through various biochemical and functional links in order to maintain host homeostasis and health. As mentioned previously, the GM directly communicates with the central nervous system (gut–brain axis) [24,25,26,27,28]. Probiotic supplementation can create a healthy gut environment in the host by balancing bacterial populations and stimulating favorable metabolic actions [54,55]. The probiotic microbe EcN has been extensively investigated for its ability to serve as a suitable agent for the intervention of various human diseases, including infectious diseases, without causing any adverse effects on human health [56,57,58]. In the present study, EcN supplementation was shown to alleviate streptomycin-induced anxiety, indicating that it exerted psycho-protective actions via microbiota modulation. It has also been reported that several conventional probiotics significantly reduce anxiety and depressive symptoms [59,60,61]. For example, supplementation with a probiotic sachet containing two strains of *Lactobacillus helveticus* and *Bifidobacterium longum* resulted in a significant improvement in the mental health of healthy women [62]. Furthermore, the administration of the probiotic *B. infantis* ameliorates the symptoms of depression by elevating the plasma tryptophan levels and decreasing the serotonin and dopamine metabolite levels in the frontal cortex and amygdaloid cortex, respectively [63]. Although several conventional probiotic microbes have been known to display anti-anxiety or anti-depressive actions [5,59,60,61,64], the present study is the first report on the effects of EcN against antibiotic-induced adverse outcomes in the psychological system.

In addition to its effects on anxiety-linked behavior, EcN administration retarded body weight gain. Although the link between anxiety and body weight involves a complex network, previous studies have reported that depression and anxiety disorders are potently associated with body weight changes [45,46]. A recent study demonstrated that anxiety can reduce body weight via increased energy expenditure by activating the adaptive thermogenesis of adipose tissues and basal metabolism through sympathetic nervous system activation [47]. This study indicated that GABAergic (gamma-aminobutyric acid-ergic) neurons are a link between anxiety and metabolic dysfunction since mice with elevated anxiety due to impaired GABAergic transmission were considerably lean and resistant to diet-induced obesity because of an increase in their basal metabolic rate and the levels of thermogenesis. In our study, mice with streptomycin-induced anxiety (those from the ST group) displayed marginal weight gain inhibition. Even though 10 days are a very short time to evaluate body weight comparison, we observed a decreasing tendency in body weight. Further studies are required to investigate the relationship between streptomycin-induced anxiety and body weight changes. However, EcN administration significantly prevented weight gain in C57BL/6 mice. The GM composition is a crucial contributor to metabolic disorder susceptibility, including obesity and eating disorders. Probiotic treatment can restore the disturbance of the microbiota community, leading to the promotion of gut health and metabolic improvement [5,64,65,66]. Moreover, EcN has been found to improve microbiota community composition under conditions of metabolic stress [67]. EcN administration can cause differences in psychological status (appetite) through the regulation of the gut–brain axis. Moreover, EcN reduces food intake and increases energy expenditure in mice fed with a high-fat diet [67], which can account for the retardation of body weight gain in the present model via appetite suppression in individuals fed with a normal diet.

In conclusion, EcN treatment prominently counteracted streptomycin-induced anxiety in mice, along with beneficial metabolic effects, such as the acute retardation of body weight gain. These actions may be associated with the well-known action of EcN whereby it restores the microbiota community, given that dysbiotic insult is a crucial factor driving neurological changes. Otherwise, EcN directly acts on the improvement of the psychological status, independent of microbiota modulation, potently altering the neuroendocrine hormone systems. As a promising intervention, EcN treatment can facilitate psychological restoration under conditions of dysbiotic stress in antibiotic-treated subjects. The present model simulates the psychiatric risk during exposure to anti-infective agents including aminoglycoside antibiotics. Extensive evaluations are warranted in other diverse antimicrobial agent-linked psychiatric disorders. Despite the beneficial actions of EcN against streptomycin-induced anxiety-like behaviors, the small-sized present investigation has a limitation to get to the overall conclusions for anxiety-reducing capacities of probiotic EcN. Moreover, addressing the systemic and molecular mechanisms underlying the action of EcN in the gut-brain axis via future studies is necessary.

## Data Availability

The data presented in this study are available on request from the corresponding author.
